# Assessing the relation between alcohol consumption and risk of disease and mortality – reply

**DOI:** 10.1186/s12937-021-00715-3

**Published:** 2021-06-28

**Authors:** Xinyuan Zhang, Shouling Wu, Xiang Gao

**Affiliations:** 1 Department of Nutritional Sciences, The Pennsylvania State University, University Park, PA USA; 2 Department of Cardiology, Kailuan General Hospital, Tangshan, Hebei China

In response to our study on the association between alcohol consumption and risk of cardiovascular disease, cancer, and mortality [1], Alam et al. raised concerns on three specific aspects of the study, including confounding factors, exposure assessment, and the follow-up duration.

First, all participants in our study are Chinese. The reference paper cited by Alam et al. only compared differentials in heart disease mortality across black, white, Hispanic, and Asian populations [2], therefore it cannot translate directly to our study. The Kailuan study is located in Hebei Province, where 95.8% of the population are Han Chinese, according to the 2010 Population Census of China [3]. Because the proportion of ethnic minorities in this population is small and no strong evidence supports an association between specific ethnic groups in Hebei Province and different disease risks, we did not adjust for ethnicity in our study. Further, all participants dwelled in a traditional Chinese industrial community, with relatively homogeneous lifestyles. Although generalization to other populations might be limited, it could greatly reduce potential residual confounding and enhance internal validity.

Second, the amount of alcohol was calculated using the self-report intake of a specified type and portion size of alcoholic beverages. This is a commonly used approach for estimation of alcohol intake, which has been used and validated in previous studies [4, 5]. Specifically, the self-report alcohol intake has previously been validated against blood high-density lipoprotein cholesterol concentration in our cohort [6]. However, we agree that more detailed information, eg., the brand of the beverage, could be collected to improve the estimation of alcohol intake.

Third, we agree with Alam et al. that one of the potential limitations of this study is a relatively short follow-up duration (that is ~ 10 years). Because the Kailuan Study is an ongoing prospective cohort, we will update analyses in the future when appropriate.
